# Community-based management versus traditional hospitalization in treatment of drug-resistant tuberculosis: a systematic review and meta-analysis

**DOI:** 10.1186/s41256-016-0010-y

**Published:** 2016-08-02

**Authors:** Abimbola Onigbanjo Williams, Olusesan Ayodeji Makinde, Mojisola Ojo

**Affiliations:** 1 School of Public Health, Rutgers, The State University of New Jersey, 683 Hoes Lane, Piscataway, New Brunswick, 08854 NJ USA; 2 Ernest Mario School of Pharmacy, Rutgers, The State University of New Jersey, Piscataway, New Brunswick, NJ USA; 3 Viable Knowledge Masters, 22 Olusegun Obasanjo Street, Peace Court Estate, Lokogoma, Abuja Nigeria; 4 Demography and Population Studies Program, Schools of Public Health and Social Sciences, University of the Witwatersrand, Johannesburg, South Africa

**Keywords:** Multidrug resistant tuberculosis, Extensively drug resistant tuberculosis, Community-based treatment, Hospitalization, MDR-TB high burden countries

## Abstract

**Background:**

Multidrug drug resistant Tuberculosis (MDR-TB) and extensively drug resistant Tuberculosis (XDR-TB) have emerged as significant public health threats worldwide. This systematic review and meta-analysis aimed to investigate the effects of community-based treatment to traditional hospitalization in improving treatment success rates among MDR-TB and XDR-TB patients in the 27 MDR-TB High burden countries (HBC).

**Methods:**

We searched PubMed, Cochrane, Lancet, Web of Science, International Journal of Tuberculosis and Lung Disease, and Centre for Reviews and Dissemination (CRD) for studies on community-based treatment and traditional hospitalization and MDR-TB and XDR-TB from the 27 MDR-TB HBC. Data on treatment success and failure rates were extracted from retrospective and prospective cohort studies, and a case control study. Sensitivity analysis, subgroup analyses, and meta-regression analysis were used to explore bias and potential sources of heterogeneity.

**Results:**

The final sample included 16 studies involving 3344 patients from nine countries; Bangladesh, China, Ethiopia, Kenya, India, South Africa, Philippines, Russia, and Uzbekistan. Based on a random-effects model, we observed a higher treatment success rate in community-based treatment (Point estimate = 0.68, 95 % CI: 0.59 to 0.76, *p* < 0.01) compared to traditional hospitalization (Point estimate = 0.57, 95 % CI: 0.44 to 0.69, *p* < 0.01). A lower treatment failure rate was observed in community-based treatment 7 % (Point estimate = 0.07, 95 % CI: 0.03 to 0.10; *p* < 0.01) compared to traditional hospitalization (Point estimate = 0.188, 95 % CI: 0.10 to 0.28; *p* < 0.01). In the subgroup analysis, studies without HIV co-infected patients, directly observed therapy short course-plus (DOTS-Plus) implemented throughout therapy, treatment duration > 18 months, and regimen with drugs >5 reported higher treatment success rate. In the meta-regression model, age of patients, adverse events, treatment duration, and lost to follow up explains some of the heterogeneity of treatment effects between studies.

**Conclusion:**

Community-based management improved treatment outcomes. A mix of interventions with DOTS-Plus throughout therapy and treatment duration > 18 months as well as strategies in place for lost to follow up and adverse events should be considered in MDR-TB and XDR-TB interventions, as they influenced positively, treatment success.

## Background

Multidrug resistant tuberculosis (MDR-TB) and extensively drug resistant tuberculosis (XDR-TB) have emerged as significant public health threats and pose a significant risk to the control of tuberculosis (TB) worldwide [1]. Globally, an estimated 3.3 % of new TB cases and 20 % of previously treated cases become multidrug resistant [1]. In 2014, there were about 480,000 new cases of MDR-TB worldwide and approximately 190,000 deaths from the disease. An estimated 9.7 % of people with MDR-TB have XDR-TB [1].

Treatment for drug resistant TB patients has focused on hospital and ambulatory based management [2–4]. The rationale for this has been to monitor complex drug regimens, optimize adherence, and limit community transmission [5]. Centralized interventions can be advantageous, as they concentrate on MDR-TB cases from larger regions and allow for management by trained experts in low-prevalence settings [6]. Despite the effectiveness of centralized interventions, hospital and ambulatory based management have limitations such as the need for monthly follow up visits, increased economic and social costs involved in keeping patients isolated in hospitals and long waiting lists of TB patients needing treatment [7–10]. Additionally, inadequate human resources to deliver treatment and care, insufficient bed capacity to hospitalize all new patients, and the difficulty in retaining and monitoring patients on discharge at the end of the intensive phase [7–10] have contributed to poor treatment success rates and increasing lost to follow up. Due to limited healthcare resources and long-term treatment regimens, community-based treatment has been utilized as an alternative care model to hospital-based treatment [10–12].

Community-based management of MDR-TB incorporates two key strategies: decentralization of hospital care from a distant specialist unit to a local district hospital and early discharge of MDR-TB patients into the community. Decentralization is achieved by the development of infrastructure for in-patient care at a district level hospital and skills transfer from the specialist referral unit [13]. Several studies [5, 6, 14] have utilized a mix of interventions for community management of drug resistant tuberculosis (DR-TB) treatment, which includes utilization of family members and healthcare workers to administer DOTS-Plus, social assistance, support groups, routine home visits, and clinician support at the community-based sites.

Findings from several studies [10, 15–17] suggest the impact of community-based treatment to be more effective than care in a traditional hospitalization setting, grounded on improved treatment success rate, lower lost to follow up, and shorter time to treatment initiation. Also, community-based treatment has been shown to increase access to care by improving access to diagnostic and treatment services to further strengthen treatment success rate [10–12]. The delivery of community-based TB treatment through community health workers has further improved access and service utilization of healthcare [10–12]. Thus, community-based treatment has facilitated access to treatment by making treatment closer to patient’s home, and enhancing support to patients and their families. Community-based management of MDR-TB is considered vital and cost effective [18] especially in low resource settings.

So far, only one meta-analysis has focused on comparing community-based treatment to hospitalized treatment for DR-TB [19]. Although, another study (not a meta-analysis) has compared treatment outcomes in community-based care versus centralized hospitalization in South Africa [15], however, more evidence is required in determining the influence of community-based treatment in bringing about increased treatment success rate. We carried out a systematic review and meta-analysis to compare the effectiveness of community-based management to traditional hospitalization in the care of patients with MDR-TB and XDR-TB patients in the 27 MDR-TB HBC.

## Methods

### Search strategy and selection criteria

We searched PubMed, Cochrane, Lancet, and Centre for Reviews and Dissemination (CRD), Web of Science, and the International Journal of Tuberculosis and Lung Disease for studies published from January 2005 to October 2015. Searches were done from September 1, 2015 to October 31, 2015. The search included MeSH terms for MDR-TB and XDR-TB, “community DOTS-Plus” or “community health services” or “decentralized” or “home based care” and “hospitalization” or “centralized” or “in patient”.

We included retrospective cohorts, prospective countries, and a case control study implementing community-based and hospitalized treatment in MDR-TB and XDR-TB patients aged >18 years in the 27 MDR-TB HBC. Studies were included if they were published in English language, had at least ten patients in each study group, and patients were treated for a minimum of six months. For authors having more than one article on the topic, the most recent article was accepted or if the content was found different after review, then the other studies were also accepted. Studies were excluded if the study design was cross sectional or qualitative or did not report data that were useful for extraction. Studies with a sample size < 10, active TB cases, utilized surgical interventions, and exclusively used first line therapy in their treatment protocol were also excluded.

Studies were considered community-based if the model of care was implemented in a decentralized setting, utilized family members, and local healthcare workers to directly observe treatment. For traditional hospitalization, our selection criteria included implementation in a centralized setting, and treatment requiring hospitalizations or frequent visits to a healthcare facility.

### Screening and data extraction

**Fig. 1:**
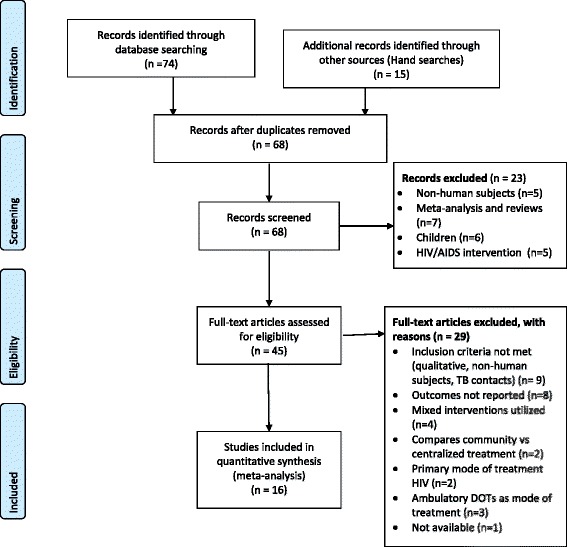
PRISMA Study flowchart

Titles and abstracts of all articles were screened and retrieved by AOW and MO to identify potentially eligible studies. AOW and MO reviewed the full text of potentially eligible articles. These were evaluated using the inclusion and exclusion criteria. Database search results were imported into PubMed Bibliography, ProQuest Flow, and duplicate records were removed. Reasons for the exclusion of studies are documented and presented in Fig. 1. Information on the study characteristics and the primary treatment outcome of interest (treatment success rate or treatment failure rate) was extracted into Systematic Review Data Repository (SRDR) tool. Additionally, information on relevant treatment characteristics (adverse rate, default rate, regimen model, regimen duration, location, and provider) was extracted. These outcomes were only extracted when provided in studies.

Treatment failure rate was defined as the proportion of patients that failed MDR-TB or XDR-TB treatment, whereas treatment success rate was defined as the proportion of patients that completed MDR-TB or XDR-TB treatment and cured. Adverse rate was defined as the proportion of patients who suffered an adverse event or if a medical decision was made to terminate treatment due to poor response. Lost to follow up was defined as the proportion of patients that interrupted DR-TB treatment for two or more consecutive months for any reason.

To minimize potential errors, included articles and the SRDR data extraction template were reviewed repeatedly. When uncertainty arose with deciding the inclusion of studies, opinion of experts implementing DR-TB programs in Nigeria were sought. This only occurred twice throughout the duration of the study.

### Assessment of risk of bias within and across included studies

Since studies were observational, the methodological quality of each study was determined using the Newcastle-Ottawa Quality Assessment Scale (NOS) scale and the Strengthening the Reporting of Observational Studies in Epidemiology (STROBE) statement [20, 21]. In the NOS scale, a maximum of nine points is assigned to cohort studies and eight points to case control. Studies are assigned points for measurement of exposure and outcomes, and selection of participants. Studies with NOS score < 4 were deemed low quality, 4–5 as moderate quality, and ≥ 6 as high quality. Furthermore, the risk of bias across studies was assessed using the Grading of Recommendations Assessment, Development, and Evaluation (GRADE) system. Studies were classified as low (observational study), and very low (any other evidence). According to Higgins and Green [22], definitions of GRADE of evidence include;

- High: Further research is very unlikely to change confidence in the estimate of effect
- Moderate: Further research is likely to have an important impact on the confidence in the estimate of effect and may change the estimate
- Low: Further research is very likely to have an important impact on our confidence in the estimate of effect and is likely to change the estimate
- Very low quality: We are very uncertain about the estimate

Furthermore, the Cochrane Collaboration’s handbook was used to assess whether certain steps were taken to reduce the risk of bias under six domains [23]. Domains include allocation concealment, blinding, incomplete outcome data, and sequence generation, selective outcome reporting and other sources of bias. Judgment was categorized as yes (low risk of bias) and no (high risk of bias) or unclear.

### Data synthesis and analysis

Using a random effects model, a one-arm meta-analysis was conducted using Open Meta-Analyst (OMA) software to analyze and record data from the included studies [24]. Due to the nature of data from eligible studies (i.e. cases without controls or comparison groups), a one-arm meta-analysis was suitable. Thus, the odds ratio could not be used to estimate the strength of association rather the use of proportion was suitable. Meta-analysis was performed by analyzing separately and comparing studies classified as community based and hospitalization that reported outcomes on treatment success and treatment failure.

Data are presented graphically using the forest plot, in which the proportion, its 95 % CI, and the overall summary statistic was estimated [25]. Under the assumption of the random effects model, estimates of tau square (tau^2^), Q statistic, and I square statistic (I^2^) were generated and used to evaluate heterogeneity [25]. An I^2^ value of 50 % or greater and where *P* < 0.05 was used to denote high heterogeneity [26]. Meta-regression analysis was conducted to assess whether effect estimates differed by patient age, adverse effect, default rate, and treatment duration, thus explaining any of the heterogeneity in the study [25, 27]. The dependent variable was DR-TB success rate and all 9 studies that implemented community-based treatment were included. The analysis was based on a significance at alpha 0.05 level.

Sensitivity analysis using trim and fill was conducted to further explain the heterogeneity observed [28]. The potential for publication bias was considered by assessing a visual inspection of the funnel plot symmetry, Begg’s regression, and Egger linear regression test. Additionally, subgroup analysis was conducted to investigate heterogeneity and determine if outcomes differ on several study and intervention characteristics. The Preferred Reporting Items for Systematic Reviews and Meta-Analyses (PRISMA) statement was adhered to in this review [29].

## Results

Our search identified a total of 89 publications, of which 21 were duplicates (Fig. 1). Of the 68 articles screened, 23 articles were not eligible and excluded. Full text of 45 articles was reviewed, of which 29 articles were excluded due to the following reasons: qualitative study design, meta-analysis review, mode of treatment, and desired outcomes not reported. One study was not found because it was not available on CRD as at the time of the search. In total 16 studies from 9 MDR-TB HBC (Bangladesh, China, Ethiopia, Kenya, India, South Africa, Philippines, Russia, and Uzbekistan) met the inclusion criteria for this study.

### Study characteristics

Table 1 shows the summary characteristics of the 16 studies included in the meta-analysis. Nine studies [6, 11, 14, 30–35] implemented a community-based intervention while seven studies [36–42] implemented traditional hospitalization. The sample size for each category of patients includes: XDR-TB (29), MDR-TB & XDR-TB (807) and MDR-TB (2508). Twelve studies [6, 11, 14, 31, 33–36, 39–42] included MDR-TB patients, three studies [30, 32, 37] had both MDR and XDR TB patients, and one study [38] included only XDR-TB patients.

**Table 1:** Summary of Findings: Community-based treatment compared with traditional hospitalization for MDR-TB and XDR-TB patients

Author, Year, Country of Study, Study period	Arm, N, Percent Female, Age	Intervention Components	Intervention setting, Intervention provider, Length of DOTS	Drug model, number of drugs, treatment duration (intensive, continuation phase), Proportion previously treated
Brust JC 2012 [12] Prospective cohort South Africa 2008–2010	Community-based intervention *N* = 80 Female: 63 % Age: 34 HIV+: 83 %	Extensive training of PHC staff, Routine home visits, Clinician support, DOTS supervised by healthcare worker, DOTS supervised by family treatment supporter, DOTS supervised by a healthcare worker, Education of patients and family treatment supporter, Adherence support and adverse event monitoring, Mobile multidisciplinary teams of home care providers & HIV treatment	Decentralized, outpatient Friends/relatives staying close by, Home care support, DOTS nurse, Community Health Extension Worker (CHEW) Throughout therapy	Standardized 6, NR 6, 24 NR
Brust JC 2010 [39] Retrospective cohort South Africa 2000–2003	Traditional hospitalization *N* = 1209 Female: 39 % Age: 33 HIV+: 52 %	Hospitalization	Hospital No DOTS provider Partial Observation	Standardized 5,4 4-6, 18 NR
Cox H 2007 [41] Retrospective cohort Uzbekistan 2003–2005	Traditional Hospitalization *N* = 87 Female: 39.1 % Age: 31 HIV+: NR	Hospitalization	Hospital Trained facility based healthcare worker NR	Individualized 6, NR 6,18 100 %
Cox H 2014 [30] Retrospective cohort South Africa 2005–2011	Community-based intervention *N* = 1208 Female: 50 % Age: 33 HIV+: 70 %	Extensive training of primary health care center (PHC) staff, Routine home visits, Clinician support, Social assistance and support groups, DOTS^a^ supervised by healthcare worker	PHC Trained facility based healthcare worker Throughout therapy	Standardized 5, NR ^b^ 6, 18 NR
Hirpa S 2013 [42] Case control study Ethiopia 2011–2012	Traditional Hospitalization *N* = 134 Female: 39.5 % Age: 25.1 HIV+: 13.4 %	Clinician support Healthcare workers	Hospital Trained facility based healthcare worker Partial observation	Standardized 5, NR NR, NR NR, NR
Joseph P 2011 [33] Prospective cohort India 2006–2007	Community-based intervention *N* = 38 Female: 34.2 % Age: NR HIV+: NR	Extensive training of PHC center staff, Routine home visits, education of patients and family treatment supporter, Supply of drugs to local health center	NR Trained facility based healthcare worker, Friends/relatives staying close by, Private medical practitioners Throughout therapy	Standardized 6,4 6–9, 18 NR
Keshavjee S 2008 [38] Retrospective cohort Russia 2000–2004	Traditional Hospitalization *N* = 608 Female: NR Age: 33.9 HIV+: NR	Hospitalization and DOTS supervised by healthcare worker	Hospital DOTS supervised by healthcare worker Partial Observation	Individualized 5, 5 6–9, 18 100 %
Liu CH 2011 [37] Retrospective cohort China 1996–2009	Traditional Hospitalization *N* = 576 Female: 33.9 % Age: 41 HIV+: NR	Clinician support	Hospital NR NR	Individualized 5, NR ^c^ 18, 18 68.7 %
Vaghela JF 2015 [14] Prospective cohort India 2009–2012	Community-based intervention *N* = 101 Female: 40.6 % Age: 33 HIV+: NR	Extensive training of primary health care center staff, Physical and mental support Counseling, Routine home visits, Adherence support and adverse event monitoring, Mobile multidisciplinary teams of home care providers, Vocational rehabilitation, Hygiene & Nutrition counseling, Nursing care, Financial rehabilitation	PHC, Patient home Trained facility based healthcare worker, Home care support Throughout therapy	NR NR, NR 6, 24–27 NR
Oyieng’o D 2012 [11] Retrospective cohort Kenya 2008–2010	Community-based intervention *N* = 14 Female: 50 % Age: NR HIV+: 50 %	Extensive training of PHC staff, Routine home visits, Clinician support, DOTS supervised by family treatment supporter, DOTS supervised by healthcare worker, Education of patients and family treatment supporter, Adherence support and adverse event monitoring, Mobile multidisciplinary teams of home care providers	Decentralized, Local Health Centre Friends/relatives staying close by, Home care support, DOTS nurse, CHEW Throughout therapy	Standardized 5,4 6, 18 NR
Singla R 2009 [34] Retrospective cohort India 2002–2006	Community-based intervention *N* = 126 Female: 42 % Age: 26 HIV+: NR	DOTS supervised by family treatment supporter, Daily supervised treatment in peripheral health centers, decentralized care	Decentralized Friends and family staying close by Throughout therapy	Standardized 5,3 6–9, 18 NR
Shin SS 2007 [40] Retrospective cohort Russia 2000–2002	Traditional Hospitalization *N* = 244 Female: 9.2 % Age: 31 HIV+: NR	Hospitalization and trained facility based healthcare worker	Trained facility based healthcare worker Throughout therapy	Individualized NR, NR 18.5, 18 100 %
Tupasi TE 2006 [31] Retrospective cohort Philippines 1999–2002	Community-based intervention *N* = 117 Female: 26 % Age: 38 HIV+: NR	DOTS supervised by healthcare worker, Daily supervised treatment in peripheral health centers, Home based DOTS	PHC, Patient home Trained facility based healthcare worker Partial Observation	Individualized NR, NR 6, 18 18.8 %
Thomas A 2007 [32] Prospective cohort India 1999–2003	Community-based intervention *N* = 66 Female: 30.3 % Age: 38 HIV+: NR	Routine home visits, Clinician support, DOTS supervised by healthcare worker, Financial rehabilitation	PHC, Patient home Trained facility based healthcare worker, Village health worker, private provider Partial Observation	Individualized 5,NR 6–9, 12 100 %
Van Deun A 2010 [36] Prospective cohort Bangladesh 1997–2007	Traditional hospitalization *N* = 427 Female: 25.5 % Age: 31.7 HIV+: NR	Clinician support	Hospital Trained facility based healthcare worker NR	Standardized NR, NR NR, NR 100 %
Wei X 2015 [35] Retrospective cohort Ethiopia 2990–2102	Community- based intervention N: 110 Female: 26.4 % HIV+: Yes (NR)	Routine home visits, DOTS supervised by healthcare worker and family	PHC, Patient home Village health worker, family Throughout therapy	Standardized 5, NR 6, 18 NR

^a^DOTS, Directly observed therapy short course

^b^ *NR* Not reported

^c^ *NR* Not reported

Eight studies [30–32, 36, 38, 40–42] utilized healthcare workers, two studies [6, 11] utilized a combination of home care support teams and families, two studies [33, 35] used both healthcare workers and family, and one study each used home care support teams (14) and family [34] as a DOTS-Plus provider. For treatment of DR-TB patients, nine studies [6, 11, 30, 31, 33–36, 39, 42] utilized standardized treatment and six used individualized regimen [31, 32, 37, 38, 40, 41]. Treatment duration for the intensive and continuous phase ranged from 4 to 6 months and 12–27 months. Eleven studies [11, 30–32, 34, 35, 37–39, 42] used at least five drugs and two studies [6, 33] used six drugs in their treatment regimen. In addition, DOTS-Plus was observed throughout therapy in 9 studies [6, 11, 14, 30, 33–36, 40] and five studies [31, 32, 38, 39, 42] reported partial observation.

### Assessment of risk of bias in individual studies

Table 2 provides a detailed overview of items evaluated against each study using the NOS statement, STROBE, GRADE methodology, and Cochrane domains to assess the risk of bias within and across studies. Six studies [36–40, 42] were evaluated as having low quality and the rest of the studies have very low quality. Only four studies [6, 40–42] had incomplete outcome data and six studies [6, 31, 33, 35, 41, 42] have selective outcome reporting.

**Table 2:** Assessment of risk of bias within and across included studies

Study	Study year	NOS/ STROBE score	GRADE	Allocation concealment (Selection bias)	Blinding	Incomplete outcome data	Random sequence generation	Selective outcome reporting	Other sources of bias
Cox H	2014	4/19	VL	N	Y	Y	N	Y	A
Brust JC	2012	4/19	VL	N	Y	N	N	N	A, D
Vaghela JF	2015	4/19	VL	N	Y	Y	N	Y	A, D
Oyieng’o D	2012	4/19	VL	N	Y	Y	N	N	A, D
Joseph P	2011	4/19	VL	N	Y	Y	N	N	A, D
Van Deun A	2010	5/20	L	N	Y	Y	N	Y	A, D
Brust JC	2010	5/20	L	N	Y	Y	N	Y	A, D
Singla R	2009	4/19	VL	N	Y	Y	N	Y	A, D
Tupasi TE	2006	4/19	VL	N	Y	Y	N	Y	A, D
Thomas A	2007	4/19	VL	N	Y	Y	N	Y	A, D
Liu CH	2011	5/20	L	N	Y	Y	N	Y	A, D
Keshavjee S	2008	5/20	L	N	Y	Y	N	Y	A, D
Shin SS	2007	5/19	L	N	Y	N	N	Y	A, D
Cox HS	2007	4/19	VL	N	Y	N	N	N	A,D
Wei XL	2015	4/19	VL	N	Y	Y	N	N	A,D
Hirpa S	2013	5/20	L	N	Y	N	N	N	A, D

A Attrition bias, D Detection bias

VL Very Low: We are very uncertain about the estimate

L Low: Further research is very likely to have an important impact on our confidence in the estimate of effect and is likely to change the estimate

H High: Further research is very unlikely to change our confidence in the estimate of effect

NOS score < 4: Low quality

NOS score 4–5: Moderate quality

Y: Low risk of bias

N: High risk of bias

### Results of individual studies

**Fig. 2:**
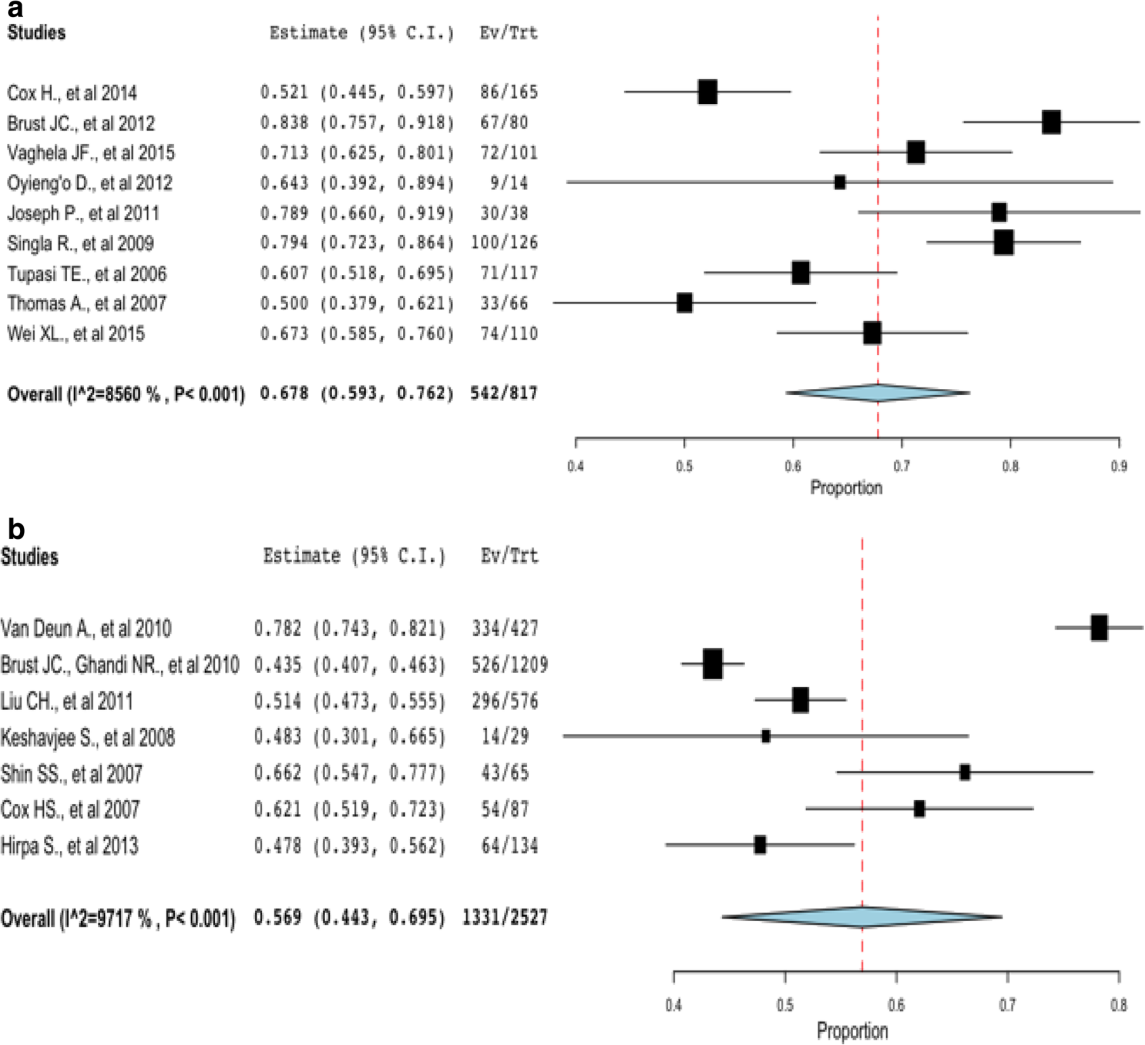
Pooled treatment success rate (cured and treatment completed) of MDR-TB and XDR-TB community-based intervention versus traditional hospitalization. The pooled treatment success rate for community-based studies (**a**) is higher than studies that utilized the traditional hospitalization (**b**) for treatment of MDR-TB and XDR-TB cases

In Fig. 2, five studies [11, 30–32, 35] implementing community-based treatment had treatment success rate less than the overall summary estimate of 67.8 % and four studies [37–39, 42] utilizing traditional hospitalization had treatment success rate less than the overall treatment success rate of 56.9 %. The p value for heterogeneity was <0.001, with I^2^ = 85.60 %, indicating a significant heterogeneity among studies. Four studies [31–33] reported treatment failure rates higher than the overall summary estimate of 6.5 % for community-based studies and 3 studies [37, 38, 42] utilizing traditional hospitalization had treatment failure rates above its overall estimate of 18.8 %, with significant heterogeneity (*p* = <0.001, I^2^ = 97.24 %).

### Synthesis of results

**Fig. 3:**
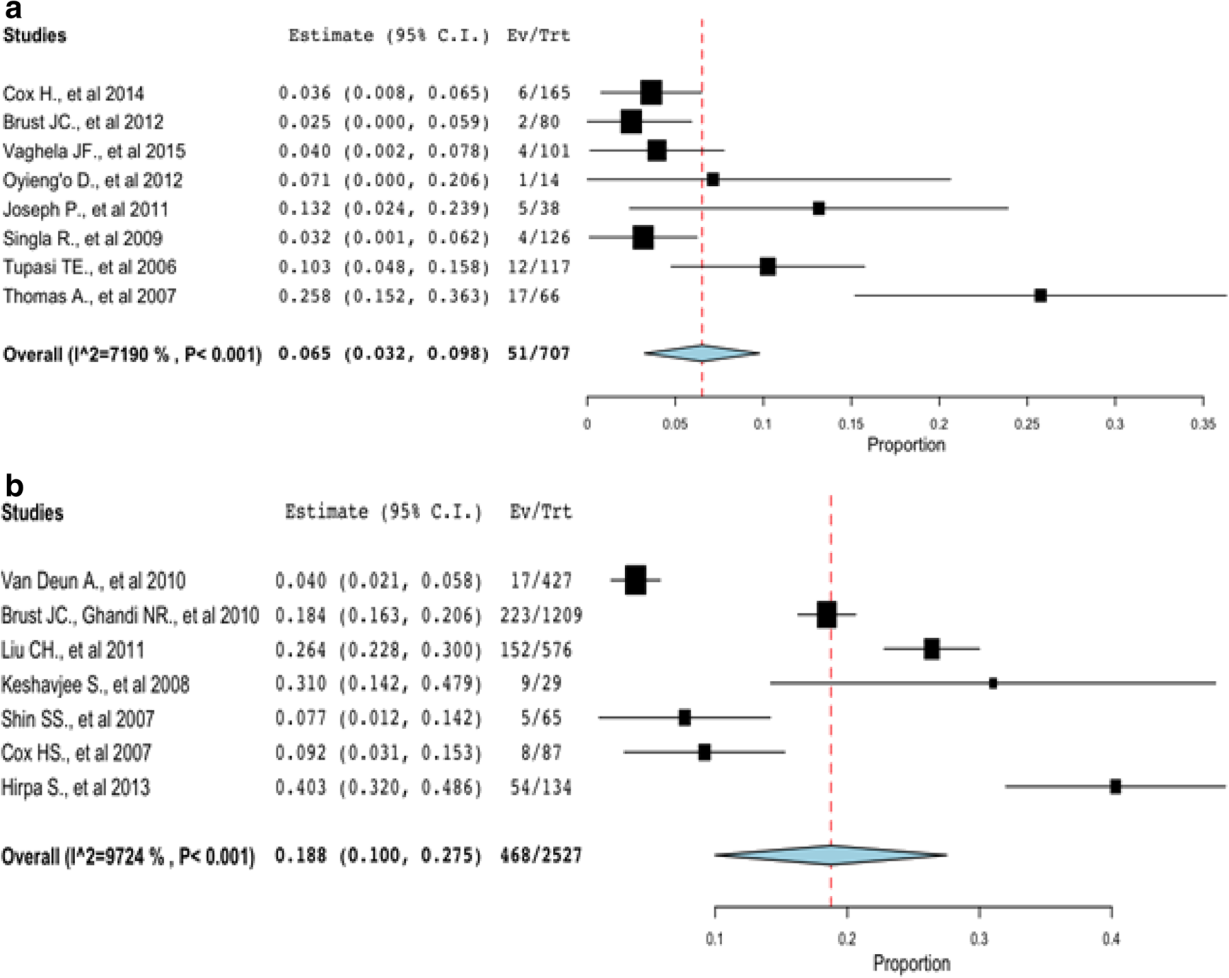
Pooled treatment failure rate of MDR-TB and XDR-TB community-based intervention versus traditional hospitalization. The pooled treatment failure rate for community-based (**a**) is lower than that of traditional hospitalization (**b**)

Pooled estimates of treatment success and failure rates were calculated as shown in Figs. 2 and 3 for community-based treatment and traditional hospitalization. The pooled probability of being cured and completing treatment using a community-based treatment is 67.8 % (95 % CI: 0.593 to 0.762) compared with traditional hospitalization at 56.9 % (95 % CI: 0.44.3 to 0.695). All studies equally contributed to the heterogeneity of the pooled estimate; thus, there was no need to investigate studies contributing to the heterogeneity.

Figure 3 shows the meta-analysis of treatment failure rate for community-based treatment and traditional hospitalization. The probability of MDR-TB and XDR-TB patients failing treatment in community-based treatment is 6.5 % (95 % CI: 0.03 to 0.10; *p* < 0.01) compared to traditional hospitalization at 18.8 % (95 % CI: 0.10 to 0.28; *p* < 0.01). There is an extremely high amount of heterogeneity of treatment failure for community-based treatment (I^2^ = 71.90 %, *p* < 0.001) and traditional hospitalization (I^2^ = 97.24 %, *p* < 0.001), where a high level of heterogeneity of I^2^ > 50 % is considered to be substantial [43].

### Subgroup analysis

Since an extremely high heterogeneity was observed, subgroup analysis of study and intervention characteristics was conducted and presented in Table 3. Studies without HIV co-infected [14, 30, 33–35] patients (72 %, 95 % CI: 0.65 to 0.79) reported higher treatment success rate than studies with HIV co-infected [6, 11, 30, 32, 35, 37–40, 42] patients (57 %, 95 % CI: 0.49 to 0.64). In addition, the 95 % CI for studies with these characteristics: duration of DOTS-plus, length of treatment, and number of drugs in regimen were non-overlapping indicating a statistically significantly relationship with treatment success. However, the 95 % CI overlapped for drug regimen model, DOTS-plus location, DOTS-plus provider, patient type, type of treated patients, adverse events, quality of studies, and start year of studies, thus, indicating studies with these characteristics are not significantly different.

**Table 3:** Results from sub group analysis on treatment success

Variables	# of Studies	Point Estimate	95 % CI	
Start year of study				
2000 or later [6, 11, 14, 30, 33–35, 38–42]	12	0.64	0.54	0.74
1999 or earlier [31, 32, 36, 37]	4	0.60	0.44	0.77
Quality of study				
Very Low [6, 11, 14, 30–35, 39, 41]	11	0.65	0.54	0.75
Low [36–38, 40, 42]	5	0.59	0.44	0.74
Adverse Events				
< 50 % [34–36]	3	0.76	0.70	0.82
> 50 % [6, 31–33, 38, 40–42]	8	0.63	0.52	0.73
Type of treated patients				
New cases and previously treated [6, 30–32, 34–37, 39, 41, 42]	11	0.61	0.52	0.71
Previously treated patients [11, 33, 38, 40]	4	0.66	0.53	0.78
HIV co-infected patients^a^				
Yes [6, 11, 30, 32, 35, 37–40, 42]	10	0.57	0.49	0.64
No [14, 30, 33–35]	6	0.72	0.65	0.79
Patient type				
MDR [6, 11, 14, 31, 33–36, 39–42]	12	0.67	0.56	0.78
MDR and XDR [30, 32, 37]	3	0.51	0.48	0.55
XDR [38]	1	0.48	0.30	0.66
DOTS-plus Provider				
Healthcare workers [30–32, 36, 38, 40–42]	8	0.59	0.48	0.69
Home care support teams & Family [6, 11]	2	0.78	0.60	0.95
Home care support teams [14]	1	0.71	0.62	0.80
Healthcare workers & Family [33, 35]	2	0.72	0.61	0.83
Family [34]	1	0.79	0.72	0.86
Drug Regimen Model				
Standardized [6, 11, 30, 33–36, 39, 42]	9	0.66	0.53	0.79
Individualized [31, 32, 37, 38, 40, 41]	6	0.57	0.51	0.62
DOTS-plus Location				
Health center [14, 30, 33, 34]	4	0.70	0.56	0.84
Patient home [6]	1	0.84	0.76	0.92
Patient home and Health center [11, 31, 32, 35]	4	0.61	0.53	0.68
Hospital [36–42]	7	0.57	0.44	0.69
Duration of DOTS-plus^a^				
Throughout therapy [6, 11, 14, 30, 33–36, 40]	9	0.72	0.65	0.79
Partial observation [31, 32, 38, 39, 42]	5	0.50	0.43	0.57
Length of treatment (months)^a^				
< 18 [32, 42]	2	0.48	0.42	0.55
18 & above [6, 11, 14, 30–35, 37, 39–41]	12	0.65	0.56	0.74
Drugs in regimen^a^				
5 [11, 30–32, 34, 35, 37–39, 41, 42]	11	0.57	0.49	0.64
> 5 [6, 33]	2	0.82	0.76	0.89

^a^Non-overlapping 95 % CI

### Meta-regression analysis

Table 4 shows the results from meta-regression analysis on continuous covariates that are independently associated with outcomes. For community-based studies, results suggest that as age of patients’ increases, treatment success rates of patients’ decreases by 3.1 % (β:-0.031, 95 % CI:-0.044 to -0.019, *p* < 0.001). We found a significant interaction between treatment success and lost to follow up (β:0.009, 95 % CI:0.005 to 0.014, *p* < 0.001). Furthermore, an increase in treatment length moderated an increase in treatment success rate (β:0.020, 95%CI:0.007 to 0.033, *p* < 0.01).

**Table 4:** Meta regression analysis of included in studies implementing community-based treatment

Variables	Coefficients	95 % CI		P-value
Age	−0.031	−0.044	−0.019	<0.001
Lost to follow up	0.009	0.005	0.014	<0.001
Adverse rate	0.005	0.003	0.006	<0.001
Treatment length	0.020	0.007	0.033	0.003

Omnibus p value: 0.000

### Sensitivity analysis and publication bias

**Fig. 4:**
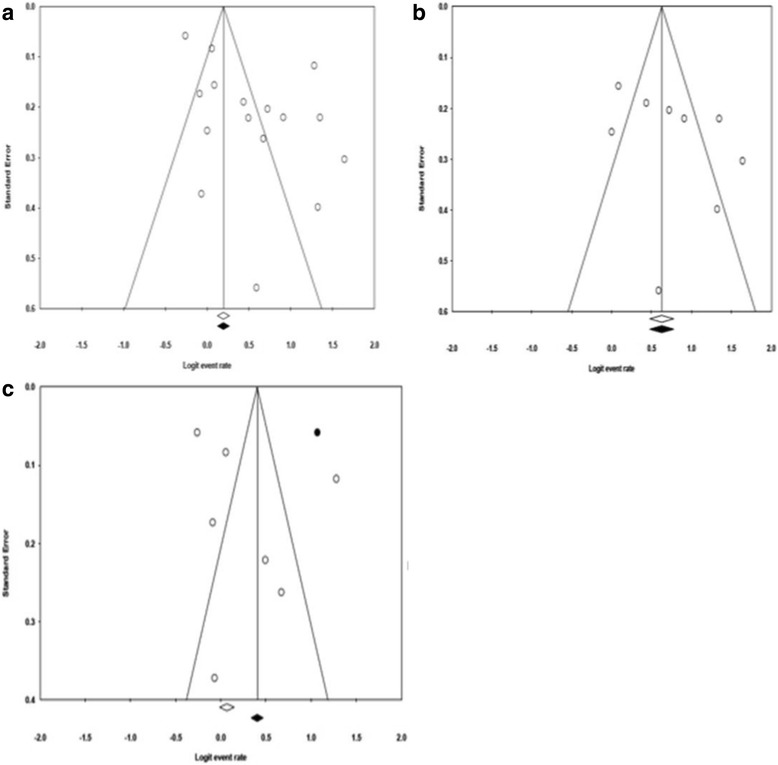
Illustration of funnel plot asymmetry due to heterogeneity. The figure shows the Funnel plot of standard error by logit event rate for all studies (**a**), community based studies (**b**), and hospital based studies (**c**)

We found conflicting results between Egger’s and Begg’s test. Egger’s test indicated asymmetrical distribution (intercept = 4.04, 95 % CI: 0.791 to 7.290, *p* = 0.018) while Begg’s adjusted rank correlation test (*p* = 0.192) did not show evidence of publication bias. A sensitivity analysis was conducted by repeating the meta-analysis using the trim and fill method to assess the effect of studies on the overall pooled estimate. In the trim and fill method, no missing study was trimmed (Point estimate = 0.634, 95 % CI: 0.557 to 0.705) (Fig. 4). Figure 4 shows funnel plot asymmetry arising from heterogeneity that is due entirely to their being distinct subgroups of studies, each with a different intervention effect. For studies implementing traditional hospitalization, one study was trimmed (Point estimate = 0.599, 95 % CI: 0.472 to 0.615) while no study was trimmed for community-based interventions (Point estimate = 0.681, 95 % CI: 0.593 to 0.685) (Fig. 4).

## Discussion

### Summary of evidence

This review combined data from 16 observational studies from 9 MDR-TB HBC. Across these studies, the overall sample size was 3344 MDR-TB and XDR-TB patients receiving second line anti-TB drugs. We compared the effect of community-based treatment with traditional hospitalization in improving treatment success rates among MDR-TB and XDR-TB patients in the 27 MDR-TB HBC. Our findings suggest that community-based treatment improved treatment success rate than traditional hospitalization methods.

All the studies reported outcomes on treatment success rate. According to the WHO Global Tuberculosis Report of 2015, only three MDR-TB high burden countries (Estonia, Ethiopia, and Myanmar) achieved a treatment success rate of ≥ 75 %, which equals the overall estimate in our study [1]. The study by Brust [12] reported the highest treatment success rate (84 %) using community-based treatment. However, with a very low quality of evidence and small sample size (*n* = 80), were having much confidence in the result may be debatable. Amongst the seven studies that implemented a traditional hospitalization method, treatment success rate ranged from 43.5 % [39] to 78.2 % [36].

Across the included studies, the pooled treatment failure estimate ranged from 6.5 % and 18.8 % for community-based and traditional hospitalization respectively. Treatment failure rates were generally lower in community-based treatments compared to traditional hospitalization treatments. We observed extremely low treatment failure rates in studies by Singla [34] and Brust [12] at 3 % and the failure rate in Keshavjee [38] at 31 % [6, 34, 38]. Our findings show a lower estimate than that obtained from the Global Tuberculosis Report [1] where 10 % of XDR-TB patients in 40 countries for whom outcomes were reported failed treatment.

Furthermore, we found that studies with significant patient and treatment characteristics significantly influenced treatment success rate. In the meta-regression model of treatment success, age, adverse rate and lost to follow up could possibly explain the extremely high heterogeneity observed, although, inferences from statistical heterogeneity may be uncertain.

Since there is a considerable high amount of heterogeneity (>75 %) between the study populations and varying sampling methods, there is a low confidence that this is the true population effect or that there even is a meaningful single effect. Statistical heterogeneity may arise because of clinical differences between studies (i.e. setting, types of participants, or implementation of the intervention) or methodological differences [44]. The extremely high heterogeneity observed from treatment outcomes studied could be as a result of combining studies with a mix of intervention components or controlling for different confounders. In addition, a plausible explanation to the high heterogeneity could be the diverse characteristics in the study settings. Despite an extensive search, studies from other HBC were not located. Likewise, differences in methodological quality may also cause heterogeneity and lead to funnel plot asymmetry. Smaller studies tend to be conducted and analyzed with less methodological rigor than larger studies [44].

### Agreements and disagreements with other studies or reviews

Treatment outcomes among patients with MDR-TB have been previously assessed [19, 45–47]. Two studies [5, 16] compared the effectiveness of centralized versus decentralized MDR-TB treatment. The treatment success rate (68 %) in this review is slightly higher than the estimate reported in the latest systematic review published in 2014, which showed a treatment success rate of 65 % in community-based treatments [19]. Furthermore, our result is slightly higher than that obtained from an individual study by Loveday et. al which obtained a treatment success rate of 58 % using community-based treatment [15].

Overall, we observed a higher treatment success rate in patients treated with standardized drug regimen than individualized drug regimen, however, this was not significantly different. Our findings are similar to that reported by Weiss [19]. On the contrary, a review by Orenstein [48] reported higher treatment success rate in patients treated with individualized regimen than standardized regimen. Furthermore, studies with duration of treatment > 18 months reported a higher treatment success rate than studies < 18 months of treatment. Our finding is similar to that from WHO guidelines for the programmatic management of DR-TB where patients previously treated with MDR regimen for a total duration of > 24 months were more successful than <24 months [47]. Although, we focused our analysis on the continuation phase, however, we found that treatment success rate was significantly higher when treatment duration was 18 months and above.

### Limitations

There are several limitations to this study. First, the pooled results of estimates may not be generalizable to all the 27 MDR-TB HBC as only 9 countries are represented in our review. Additionally, these pooled rates may not represent all MDR-TB and XDR-TB patients in sampled countries where only small sample sized studies have been done. Secondly, eligible studies utilized before and after study design, which significantly reduced the quality of our results and limited the comparability of findings. Furthermore, among the included studies, not all desired outcomes reported time points of treatment outcomes, thus limiting analysis to studies with available information.

Although, our meta-regression analysis explained heterogeneity, community-based treatment is a multifactorial intervention and other factors could interfere with treatment success, treatment failure, and high heterogeneity. Thus, the possibility of residual heterogeneity may exist [27] and it cannot be ascertained whether variables included in the model are sources of bias. The number of covariates was limited to avoid the problem of multiplicity and false-positive results [26]. However, despite these limitations it appears that community-based treatment significantly improved treatment success rates in DR-TB patients.

## Conclusion

The pooled estimate for treatment outcomes in our study indicates extremely high heterogeneity among studies, which is statistically significant. The evidence indicated that treatment success was significant among subgroups with certain study and treatment characteristics.

### Implications for practice

In this review, we examined the effects of subgroups and meta-regression on treatment outcomes. In view of the limited data on MDR-TB and XDR-TB from other MDR-TB HBC, we have identified community-based treatment to improve treatment outcomes in MDR-TB and XDR-TB patients. Our findings here further strengthen the need for decentralizing MDR-TB treatment, integrating patient centered care, and financing for TB treatment to expand community-based treatment interventions. Community-based treatment can be tailored to suit diverse settings as well as patient and treatment characteristics. Thus, TB program managers should explore implementing community-based treatment rather than traditional hospitalization in MDR-TB and XDR-TB patients.

### Implications for research

Due to poor quality of included studies, well-designed studies are needed to further establish the impact of community-based management on TB treatment outcomes. Specifically, future studies should measure and report time points of data collection on treatment outcomes and detailed description of intervention components.

## Abbreviations

CDR, Centre for Reviews and Dissemination; Community Health Extension Worker (CHEW); DOTS-Plus, Directly observed therapy short course-plus; DR-TB, Drug resistant tuberculosis; GRADE, Grading of Recommendations Assessment, Development, and Evaluation; HBC, High burden countries; I^2^, I square statistic; MDR-TB, Multidrug drug resistant Tuberculosis; NOS, Newcastle-Ottawa Quality Assessment Scale; NR, Not reported; OMA, Open Meta-Analyst; PHC, Primary Health Center; PRISMA, Preferred Reporting Items for Systematic Reviews and Meta-Analyses; SRDR, Systematic Review Data Repository; STROBE, Strengthening the Reporting of Observational Studies in Epidemiology; Tau^2^, Tau square; TB, Tuberculosis; XDR-TB, Extensively drug resistant Tuberculosis.
